# Ejecta from the DART-produced active asteroid Dimorphos

**DOI:** 10.1038/s41586-023-05811-4

**Published:** 2023-03-01

**Authors:** Jian-Yang Li, Masatoshi Hirabayashi, Tony L. Farnham, Jessica M. Sunshine, Matthew M. Knight, Gonzalo Tancredi, Fernando Moreno, Brian Murphy, Cyrielle Opitom, Steve Chesley, Daniel J. Scheeres, Cristina A. Thomas, Eugene G. Fahnestock, Andrew F. Cheng, Linda Dressel, Carolyn M. Ernst, Fabio Ferrari, Alan Fitzsimmons, Simone Ieva, Stavro L. Ivanovski, Theodore Kareta, Ludmilla Kolokolova, Tim Lister, Sabina D. Raducan, Andrew S. Rivkin, Alessandro Rossi, Stefania Soldini, Angela M. Stickle, Alison Vick, Jean-Baptiste Vincent, Harold A. Weaver, Stefano Bagnulo, Michele T. Bannister, Saverio Cambioni, Adriano Campo Bagatin, Nancy L. Chabot, Gabriele Cremonese, R. Terik Daly, Elisabetta Dotto, David A. Glenar, Mikael Granvik, Pedro H. Hasselmann, Isabel Herreros, Seth Jacobson, Martin Jutzi, Tomas Kohout, Fiorangela La Forgia, Monica Lazzarin, Zhong-Yi Lin, Ramin Lolachi, Alice Lucchetti, Rahil Makadia, Elena Mazzotta Epifani, Patrick Michel, Alessandra Migliorini, Nicholas A. Moskovitz, Jens Ormö, Maurizio Pajola, Paul Sánchez, Stephen R. Schwartz, Colin Snodgrass, Jordan Steckloff, Timothy J. Stubbs, Josep M. Trigo-Rodríguez

**Affiliations:** 1 Planetary Science Institute, Tucson, AZ USA; 2 Department of Aerospace Engineering, Department of Geosciences, Auburn University, Auburn, AL USA; 3 Department of Astronomy, University of Maryland, College Park, MD USA; 4 Physics Department, United States Naval Academy, Annapolis, MD USA; 5 Departamento de Astronomía, Facultad de Ciencias, Udelar, Uruguay; 6 Instituto de Astrofísica de Andalucía, CSIC, Granada, Spain; 7 University of Edinburgh, Royal Observatory, Edinburgh, UK; 8 Jet Propulsion Laboratory, California Institute of Technology, Pasadena, CA USA; 9 Aerospace Engineering Sciences, Colorado Center for Astrodynamics Research, University of Colorado, Boulder, CO USA; 10 Northern Arizona University, Flagstaff, AZ USA; 11 Applied Physics Laboratory, Johns Hopkins University, Laurel, MD USA; 12 Space Telescope Science Institute, Baltimore, MD USA; 13 Department of Aerospace Science and Technology, Politecnico di Milano, Milan, Italy; 14 School of Mathematics and Physics, Queen’s University Belfast, Belfast, UK; 15 INAF - Osservatorio Astronomico di Roma, Rome, Italy; 16 INAF Osservatorio Astronomico di Trieste, Trieste, Italy; 17 Lowell Observatory, Flagstaff, AZ USA; 18 Lunar and Planetary Laboratory, University of Arizona, Tucson, AZ USA; 19 Las Cumbres Observatory, Goleta, CA USA; 20 Space Research and Planetary Sciences, Physikalisches Institut, University of Bern, Bern, Switzerland; 21 IFAC-CNR, Florence, Italy; 22 Department of Mechanical, Materials and Aerospace Engineering, University of Liverpool, Liverpool, UK; 23 DLR Institute of Planetary Research, Berlin, Germany; 24 Armagh Observatory and Planetarium, Armagh, UK; 25 School of Physical and Chemical Sciences, Te Kura Matū, University of Canterbury, Christchurch, New Zealand; 26 Department of Earth, Atmospheric and Planetary Sciences, Massachusetts Institute of Technology, Cambridge, MA USA; 27 Instituto de Física Aplicada a las Ciencias y las Tecnologías, Universidad de Alicante, Alicante, Spain; 28 Departamento de Física, Ingeniería de Sistemas y Teoría de la Señal, Universidad de Alicante, Alicante, Spain; 29 INAF Osservatorio Astronomico di Padova, Padua, Italy; 30 Center for Space Science and Technology, University of Maryland, Baltimore County, Baltimore, MD USA; 31 Solar System Exploration Division, NASA Goddard Space Flight Center, Greenbelt, MD USA; 32 Department of Physics, University of Helsinki, Helsinki, Finland; 33 Asteroid Engineering Laboratory, Luleå University of Technology, Kiruna, Sweden; 34 Centro de Astrobiología (CAB), CSIC-INTA, Torrejón de Ardoz, Madrid Spain; 35 Department of Earth and Environmental Sciences, Michigan State University, East Lansing, MI USA; 36 Institute of Geology of the Czech Academy of Sciences, Prague, Czech Republic; 37 Department of Geosciences and Geography, University of Helsinki, Helsinki, Finland; 38 Dipartimento di Fisica e, Astronomia-Padova University, Padua, Italy; 39 Institute of Astronomy, National Central University, Taoyuan City, Taiwan; 40 Department of Aerospace Engineering, University of Illinois Urbana-Champaign, Urbana, IL USA; 41 Laboratoire Lagrange, Université Côte d’Azur, Observatoire de la Côte d’Azur, CNRS, Nice, France; 42 INAF - Institute for Space Astrophysics and Planetology, Rome, Italy; 43 Institute of Space Sciences (CSIC-IEEC), UAB Bellaterra, Barcelona, Spain

**Keywords:** Asteroids, comets and Kuiper belt, Astrophysical dust

## Abstract

Some active asteroids have been proposed to be formed as a result of impact events^1^. Because active asteroids are generally discovered by chance only after their tails have fully formed, the process of how impact ejecta evolve into a tail has, to our knowledge, not been directly observed. The Double Asteroid Redirection Test (DART) mission of NASA^2^, in addition to having successfully changed the orbital period of Dimorphos^3^, demonstrated the activation process of an asteroid resulting from an impact under precisely known conditions. Here we report the observations of the DART impact ejecta with the Hubble Space Telescope from impact time *T* + 15 min to *T* + 18.5 days at spatial resolutions of around 2.1 km per pixel. Our observations reveal the complex evolution of the ejecta, which are first dominated by the gravitational interaction between the Didymos binary system and the ejected dust and subsequently by solar radiation pressure. The lowest-speed ejecta dispersed through a sustained tail that had a consistent morphology with previously observed asteroid tails thought to be produced by an impact^4,5^. The evolution of the ejecta after the controlled impact experiment of DART thus provides a framework for understanding the fundamental mechanisms that act on asteroids disrupted by a natural impact^1,6^.

## Abstract (web summary)

Observations with the Hubble Space Telescope reveal a complex evolution of the ejecta produced by the Double Asteroid Redirection Test (DART) spacecraft impacting Dimorphos.

## Main

**Fig. 1:**
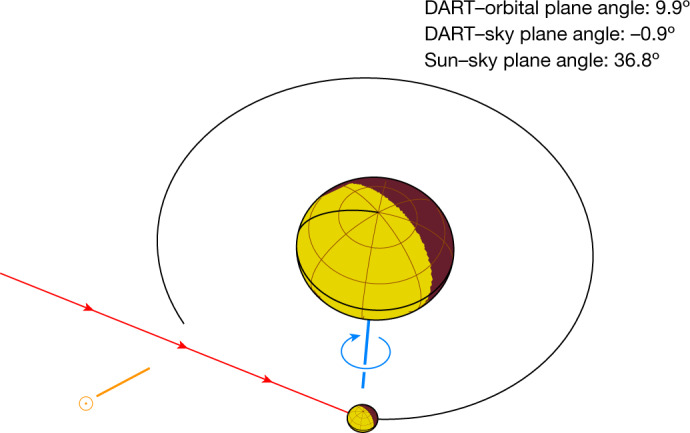
Geometry of the Didymos system at the time of impact as viewed from the HST. Sky north is in the upward direction and the east is on the left in this view. The equivalent diameters of Didymos (large spheroid) and Dimorphos (small spheroid) are 761 m and 151 m, respectively^2^. The orbit of Dimorphos around Didymos before the impact, depicted by the black circle, has a semimajor axis of 1.206 ± 0.035 km^3^ and an eccentricity of <0.03 (ref. ^29^). The sizes of Didymos and Dimorphos and their separation in the figure are to scale. The entire system lies within one pixel in the HST images. Dimorphos orbits Didymos clockwise with a speed of approximately 0.17 m s^−1^. The positive pole of Didymos (also the orbital pole of the system) is represented by the blue line, pointing close to the south celestial pole and 51º out of the sky plane away from Earth. The Sun is at a position angle of 118º, represented by the orange line and the dot-circle symbol. The DART spacecraft vector is represented by the red line, with arrows, going from east to west at a position angle of 68º and within 1º of the sky plane.

**Fig. 2:**
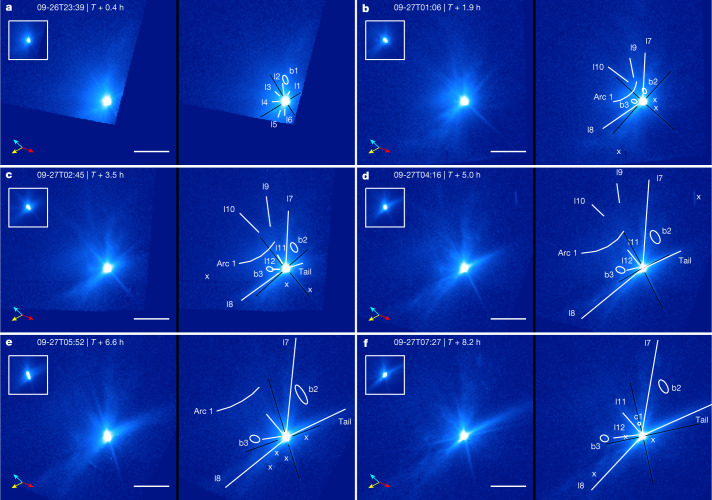
Evolution of Dimorphos ejecta from *T* + 0.4 h to *T* + 8.2 h. **a**–**f**, All images are displayed in duplicate pairs, with the left unannotated for clarity and the right annotated with features marked by white markers and labels. The inset in the top left of each panel is the 100-pixel-wide region centred on the asteroids but with the flux scaled down 10 times to show the details of the bright core. The symbol ‘x’ marks artifacts due to, for example, residual cosmic rays, frame boundaries, background objects and defective pixels. The times correspond to the mid-observation time of each image. Black lines mark diffraction spikes. All images are displayed with the same logarithmic brightness scale. Sky north is in the upward direction and the east is to the left. The yellow arrows point to the direction of the Sun, the cyan arrows the heliocentric velocity direction of Didymos and the red arrows the direction of the DART spacecraft at impact, all projected in the sky plane at the time of observation. The HST had a pointing drift during the exposures of some images, causing a smear of about 4–7 pixels in the first four images (before *T* + 5.0 h) and about 14 pixels in the *T* + 6.6 h image, all along the northeast–southwest direction (Methods). The drift widens the tail and the two diffraction spikes orthogonal to the direction of the drift. Most features are much larger than the length of the drift; we added uncertainties to account for the effect of this drift in our measurements. Many features are visible, including linear features (l1–l12), an arc (arc 1), a circular feature (c1), blobs (b1–b3) and a tail. The ejecta cone is marked by linear features l7 and l8. Scale bars are 200 km at the distance of Didymos.

The Hubble Space Telescope (HST) observed the ejecta once every 1.6 h during the first 8 h after the DART impact (Extended Data Table 1) at the viewing geometry shown in Fig. 1. The image collected at about *T* + 0.4 h (Fig. 2a) shows diffuse ejecta with several linear structures and clumps (concentration of materials ejected at similar velocities) spanning nearly the entire eastern hemisphere of Didymos. After about *T* + 2 h, the initial, diffuse dust cloud had mostly dissipated and an overall cone-shaped ejecta morphology emerged with the edges of the hollow cone shown as two linear features (l7 and l8) because of the optical depth effect. The ejecta cone showed many distinct morphological features (Fig. 2b–f), some of which are visible in several images between *T* + 3 and *T* + 10 h and extending to nearly 500 km from the asteroid. These features moved radially away from the asteroid at constant speeds of a few to about 30 m s^−1^ as projected in the sky (Extended Data Table 2). The radial expansion motion of these features suggests that this material is directly ejected from the Didymos system without being appreciably influenced by the gravity of the system or by solar radiation pressure. On the basis of the position angles (angle measured from the north towards the east) of the cone and a simple model (Methods), we find that the observed ejecta cone is consistent with a three-dimensional opening angle of 125º ± 10º and centreline at a position angle of 67º ± 8º which is almost parallel to the incoming direction of the DART spacecraft. The observed ejecta cone is wider than the ejecta produced by the vertical impact cratering experiments on granular media^7,8^, although wider opening angles could be explained by the curvature of the target surface^9^ and the angle of internal friction of the target^10^ as well as the geometry of the projectile^11^.

**Extended Data Fig. 1:**
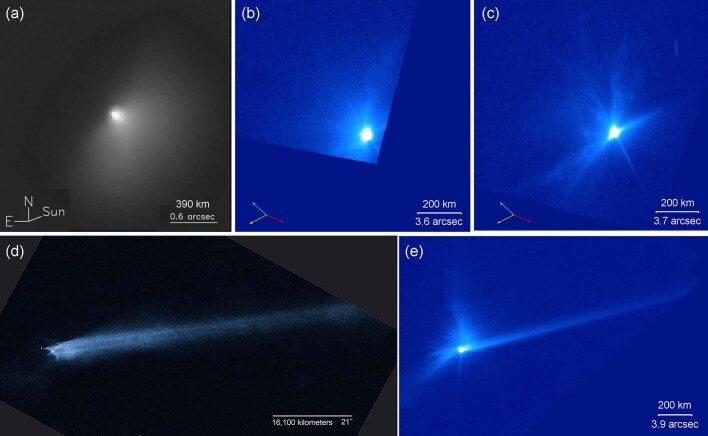
Comparison of the ejecta and tail morphology of Dimorphos with other objects. (a) Deep Impact ejecta approximately one hour after impact observed by HST^13^. (b) Dimorphos ejecta approximately T+0.4 h (Fig. 2a). (c) Dimorphos ejecta approximately T+5 h (Fig. 2d). (d) Tail of P/2010 A2 observed by HST on January 29, 2010 at a distance of 1.09 au^4^ (original image by NASA, ESA, D. Jewitt (UCLA), source: https://hubblesite.org/contents/media/images/2010/07/2693-Image.html?news=true, rotated to approximate north up). (e) Dimorphos tail observed on T+5.7 days (Fig. 4h). All images are displayed with north in the up direction and east to the left.

The ejecta of Dimorphos were different from the ejecta of comet 9P/Tempel 1 produced by Deep Impact^12^, a previous planetary impact experiment of comparable scale (Extended Data Fig. 1a–c). Both experiments delivered similar momentum to their targets. The Deep Impact spacecraft carried 80% more kinetic energy than the DART spacecraft but the 6-km diameter nucleus of Tempel 1 (ref. ^12^) was considerably more massive than the 151-m diameter Dimorphos^2^. At the scale of the HST, the Deep Impact ejecta were diffuse and mostly featureless, expanding at an average speed of about 100 m s^−1^ and a maximum speed of about 300 m s^−1^ (refs. ^13,14^). This difference in ejecta morphology is probably because of the different target compositions and subsurface structures. Whereas Tempel 1 has a highly porous subsurface^15^ composed of fine-grained dust and is rich in volatiles^16,17^, the bouldery surface and potential rubble-pile interior of Dimorphos^2^ could perturb the ejecta curtain and produce inhomogeneous structures in the ejecta^18,19^.

**Fig. 3:**
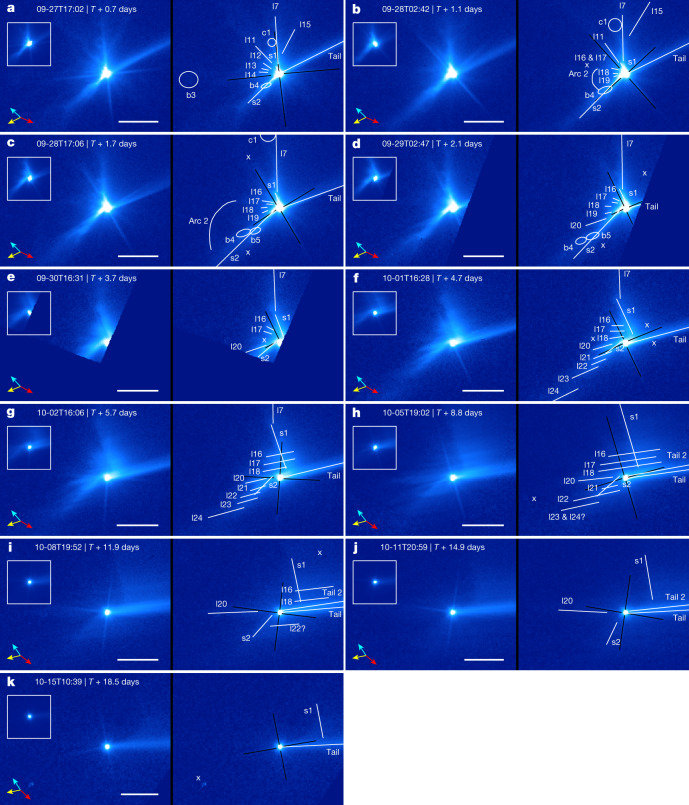
Evolution of ejecta from *T* + 0.7 days (*T* + 17.8 h) to *T* + 18.5 days. The inset, image orientation, brightness stretch, scale bars and vector arrows are all the same as in Fig. 2. The symbol ‘x’ marks imaging artifacts. The main characteristics of the ejecta during this time period include the curved ejecta streams (s1 and s2), linear features (l7, l11–l24), blobs (b3–b5), a circular feature (c1) and an arc (arc 2). **a**–**g**, The original north edge of the ejecta cone (l7) is still visible in images before *T* + 5.7 days. **a**–**e**, The early southern curved stream (s2) could be overlapped with the southern edge of the original ejecta cone (l8), which is not separately marked. **g**–**k**, The northern curved stream (s1) widened along the tail direction at about *T* + 5 days, forming a wing-like feature. **b**–**f**, A group of linear features (l16–l24), some of which are part of the southern curved stream (l21–l24), showed a clockwise rotation around Didymos from *T* + 1.1 days to *T* + 4.7 days. **g**–**i**, These linear features later (*T* + 5.7 days) stretched along the tail direction under solar radiation pressure, with those in the north of Didymos overlapping with the wing-shaped feature. **h**–**j**, A secondary tail is visible between *T* + 8.8 days and *T* + 14.9 days (also see Fig. 4). The curved edge of the wing-like feature is visible in **k**. The question marks after the annotations of l23 & l24 in **h** and l22 in **i** mark the relatively uncertain identification of these features due to their faintness and the large changes in their positions and orientations from the previous images in the sequence.

**Extended Data Fig. 2:**
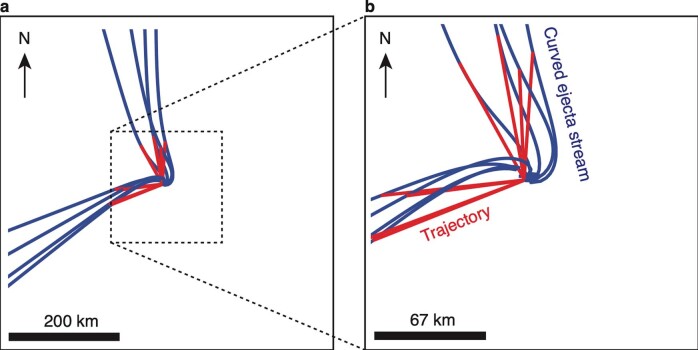
Illustration of curved ejecta streams seen by HST on T+2.1 days (Fig. 3d). (a) The red lines represent the trajectories of eight dust particles ejected at 0.43 m/s, each involved in the northern or southern edges of the ejecta cone. The initial directions are based on the measured cone geometry (Methods). The trajectories are curved by the gravity of Didymos and Dimorphos. The curved dark blue lines are the locations of several particles ejected at different speeds along the same direction as the particle in each corresponding red curve, forming the observed curved ejecta streams. The area in the illustration is 600 km wide. (b) Same illustration as (a) but with a smaller scale, showing the more remarkable curvature in the ejecta streams near the binary system. These streams capture a snapshot of particles’ positions with initial ejection speeds less than <~ 1 m/s.

From approximately *T* + 0.7 days to *T * + 2.1 days, the ejecta features composed of slower dust escaping at less than around 1 m s^−1^ emerged from the base of the ejecta cone (Fig. 3a–d). The ejecta during this stage were characterized by curved ejecta streams in the north (s1) and south (s2), some small curvilinear features (l16–l19) between them and a slight wrapping of these features around Didymos. The gravity of Didymos, which accounted for 88% of the gravitational potential of the binary system at the impact site, slowly distorted the shape of the original ejecta cone and created different morphologies for s1 and s2. The dust ejected from the original northern cone edge (l7) was in close proximity to Didymos (Fig. 1). As suggested by numerical simulation predictions^20,21^, this dust was accelerated by Didymos and the trajectories were bent before escaping the binary system, forming the northern curved stream s1 (Extended Data Fig. 2). The end of s1 near the asteroid contains relatively slow particles, the trajectories of which were bent more than those of the relatively fast particles farther away, causing the near end to shift clockwise about Didymos, resulting in an 18º twist of s1. By contrast, most of the dust in the original southern cone edge (l8; Fig. 2) was launched away from Didymos. Thus, these trajectories are less affected by the gravity of Didymos, leading to a less curved southern stream (s2) with its near end slowly wrapping around the asteroid over time (Fig. 3a–f). The small curvilinear features between the two streams (l16–l19) were probably composed of dust ejected from the front or back of the hollow ejecta cone, behaving more or less similar to either of the two curved streams and slightly rotating from the original radial direction.

Beyond the gravitational influence of the Didymos system, solar radiation pressure naturally separates particles of different sizes along the sunward–antisunward direction because small particles are accelerated faster than large particles^22^. The northern stream (s1), situated roughly orthogonal to the sunward direction, increasingly widened to form the observed wing-like shape, with a diffuse antisunward edge and a relatively sharp sunward edge (Fig. 3f–j). This sharp edge indicates a cut-off in the largest particle size of the ejecta. Because the southern stream was nearly aligned towards the Sun, those particles were first slowed by solar radiation pressure before eventually being turned towards the antisunward direction. Starting from *T* + 4.7 days, the particles moving at different speeds and directions in s2 because of the inhomogeneous distributions of dust in the ejecta were separated into individual features (l20–l24; Fig. 3f). These particles reached maximum projected sunward distances of up to 150–200 km. All of these individual features (l20–l24) and the small curvilinear features (l16–l18) between the two main streams were stretched along the sunward–antisunward direction over time by solar radiation pressure (Fig. 3f–i). The finer particles in features l16–l18, which were located to the north of Didymos, were pushed farther away from Dimorphos and caught up to the larger particles ejected into s1 earlier, appearing to overlap with the wing-like structure and creating a more complex pattern (Fig. 3g,h).

**Fig. 4:**
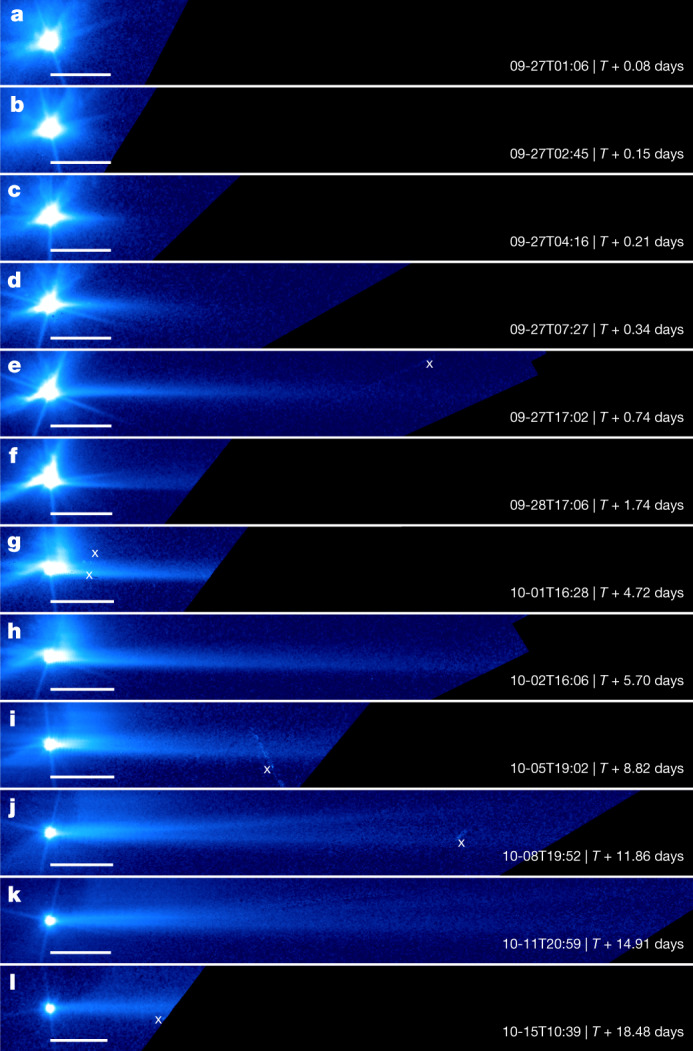
Tail formation from the Dimorphos ejecta cloud. **a**–**l**, All frames are rotated such that the expected direction of the tail based on our dust dynamic model (Methods) is in the horizontal direction extending towards the right. All frames are displayed with the same logarithmic brightness scale. The regions outside the field of view are marked by a dark blue colour. The symbol ‘x’ marks imaging artifacts. The scale bars are aligned with the asteroid at one end and extend 200 km towards the tail direction. **a–c**, Note that the first three frames have pointing-induced drift in the plane of the sky of 5–7 pixels approximately along the direction of the vertical diffraction spikes. The drift in all other frames is smaller than two pixels. The first frame (**a**) in this sequence acquired at *T* + 0.08 days (*T* + 1.9 h) shows no signs of a tail. A tail was visible starting from the second frame (**b**) acquired at *T* + 0.15 days (*T* + 3.5 h). The tail continued to grow in a direction that is, in general, consistent with an impulsive emission of dust from Dimorphos at the time of impact. **i**–**k**, The secondary tail is visible between *T* + 8.82 days and *T* + 14.91 days, pointing at about 4º north of the original tail.

**Extended Data Fig. 3:**
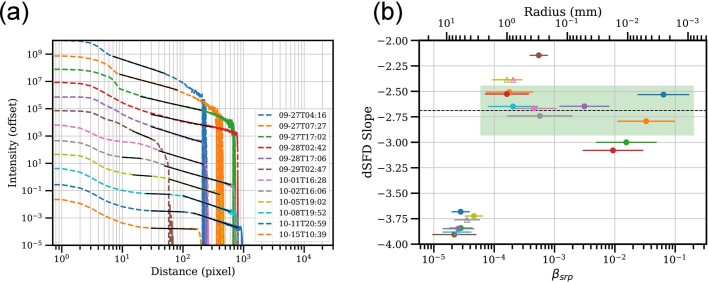
Tail brightness profile and ejecta particle size distribution. (a) Brightness profiles along the tail from various images. The dashed lines are average surface brightness extracted along the tail with a width of 40 pixels (1.6”), offset vertically for clarity. The solid lines are corresponding best-fit power law models. Two sections are fitted separately for the profiles from the images collected on and after October 2, as described in the text. (b) Best-fit power law index for the differential size distribution (dSFD) of ejecta dust particles with respect to *β*_srp_ on the bottom axis and the corresponding particle radius (assuming a density of 3500 kg/m^3^) on the top axis. Filled circles are derived from the main tail, open triangles from the secondary tail. The horizontal error bars represent the range of *β*_srp_ covered by the corresponding tail profile. The colors of symbols correspond to the colors of profiles in panel (a). The slope values from the outer section have *β*_srp_ higher than 1x10^−4^, and those from the inner section correspond to *β*_srp_ between 1x10^−4^ and 1x10^−5^. The dashed horizontal line is the average −2.7 for the outer sections, and the green shaded area represents the standard deviation.

**Extended Data Fig. 4:**
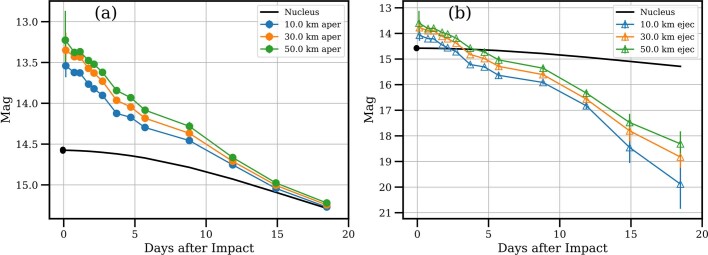
Brightness evolutions of Didymos and the ejecta. (a) Total magnitude of Didymos in 10 km, 30 km, and 50 km radius apertures at the distance of Didymos measured from HST images as a function of time after impact. (b) Magnitude of ejecta with respect to time after impact. The black curve in both panels is the magnitude of Didymos based on the IAU HG phase function model with a G = 0.20^43^, scaled to match the observed pre-impact magnitude. The ejecta magnitude corresponds to the difference between the observed total flux and the flux from Didymos. The ejecta is brighter than Didymos for about 2.5 days after impact in the 10 km radius aperture.

**Extended Data Fig. 5:**
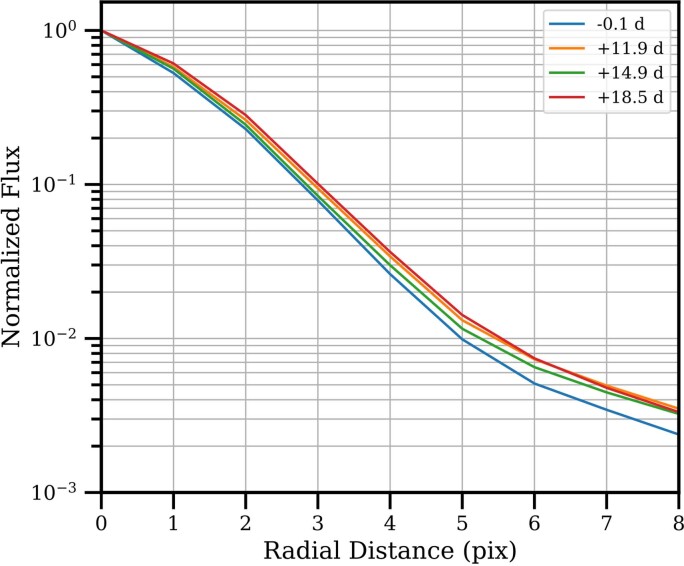
Azimuthally averaged radial profiles of Didymos and ejecta. The curves are extracted from the pre-impact image (−0.1 d) and the last three images (+11.9 d, +14.9 d, and +18.5 d). The widened PSF profiles of late images suggest a slightly extended source due to ejecta dust close to the asteroid. 1 pixel corresponds to 0.04” or 2.1–2.3 km at the distance of Didymos in the last three images.

As a result of solar radiation pressure, a dust tail started to emerge antisunward nearly opposite to the ejecta cone at about *T* + 3 h. This tail quickly stretched out to a projected length of more than 1,500  km and exceeded the spatial coverage of our images (Fig. 4). Around *T* + 5.7 days, the narrow tail showed a relatively bright and sharp southern edge and a parallel but more diffuse northern edge (Fig. 4h). The overall morphology of the tail of Dimorphos is similar to that of P/2010 A2, an active asteroid probably triggered by an impact^4,23,24^ (Extended Data Fig. 1d,e). The width of the tail, which is approximately 1 arcsecond, is consistent with an initial speed of the dust comparable with the orbital speed of Dimorphos, suggesting that the tail contains the slowest ejecta particles. Moreover, the early tail within *T* + 2 days slightly curved towards the south (Fig. 4d,e), whereas after *T* + 8 days the tail became slightly more fan-shaped (Fig. 4i–k). With radiation pressure sorting the particle size along the tail, the earliest tail at around *T* + 3 h was dominated by micrometre-sized particles, whereas centimetre-sized particles dominated the portion of the tail inside the HST field of view in the final image. The brightness profile of the tail is related to the particle-size distribution of the ejecta. Assuming a power law for the differential size distribution, we derived an exponent of −2.7 ± 0.2 for particles with radii between 1 µm and a few millimetres, and an exponent of −3.7 ± 0.2 for larger particles up to a few centimetres in radius (Extended Data Fig. 3). Ejecta particles were observed to continuously leave the Didymos system through the final images acquired after *T* + 15 days (Extended Data Figs. 4 and 5).

**Extended Data Fig. 6:**
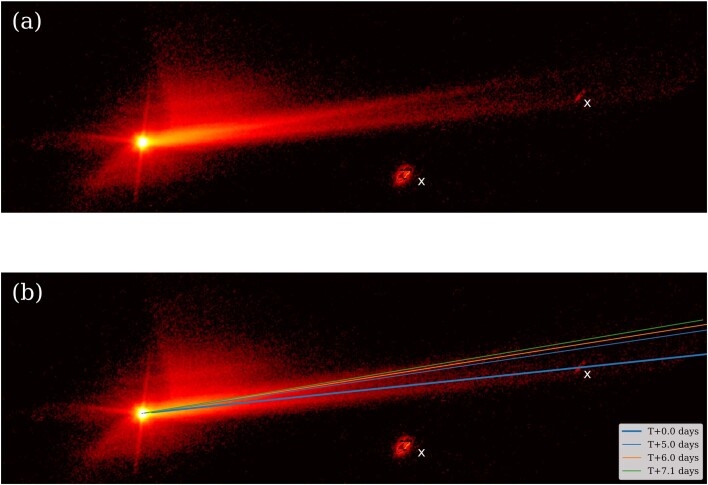
Synchrone analysis of the main tail and the secondary tail. (a) Image taken at T+11.86 days is displayed in logarithmic brightness stretch. North is up and east to the left. The features marked by “x” are artifacts from a background object and a cosmic ray hit. (b) Same image as in (a) but with synchrones corresponding to various dates overlaid. The direction of the main tail is consistent with the synchrone at impact time (T+0.0 days), and the secondary tail is consistent with the synchrones between T+5.0 and T+7.1 days.

Furthermore, a secondary tail appeared between *T* + 5.7 days and *T* + 8.8 days (Fig. 4i–k) but was no longer discernible on day *T* + 18.5 (Fig. 4l). It originated from the Didymos system and pointed about 4º north of the original tail, creating an overall fan-shaped tail morphology during this timeframe. The cause of the secondary tail is unclear, and several mechanisms will be explored (Methods and Extended Data Figs. 4 and 6), although the morphologies are consistent with the previous observations of active asteroids with multiple tails^25–28^. The whole evolutionary sequence of the ejecta of Dimorphos discussed above is shown in Supplementary Video 1.

The DART mission clearly demonstrated that impacts can activate asteroids, consistent with previous asteroid observations^1^. Our observations provide a basis for reassessing the previous observations of active asteroids thought to be triggered by an impact. The evolution of the ejecta of Dimorphos suggests that the observed particle size in active asteroid tails could depend on the age of the tail, consistent with the range of particle sizes measured in the tails of active asteroid 311P/PanSTARRS^26^. The lack of sub-millimetre-sized dust in the tail of P/2010 A2, therefore, could be a result of the observations occurring 10 months after impact^4,5,24^. DART, which is a controlled, planetary-scale impact experiment, provides a detailed characterization of the target, the ejecta morphology and the entire ejecta evolution process. DART will continue to be a model for studies of newly discovered asteroids that show activity caused by natural impacts.

## Methods

### Observations and data reduction and processing

We used a total of 19 HST orbits (period 95 min) over about 19 days to observe the Dimorphos ejecta (Extended Data Table 1). The first orbit (orbit 0o) was before the DART impact. The second orbit to the seventh orbit (orbits 01–06) started about *T* + 15 min and continuously observed the ejecta except when Earth occluded the view of the target. In the next five orbits (orbits 11–15), we observed the ejecta roughly once every 12 h, and then once every day in the subsequent three orbits (orbits 16–18). In the final phase (orbits 21–24), observations were made once every 3 days. The observations concluded 18.5 days after impact. In each orbit, images were collected at several exposure levels, in which the central core of Didymos was unsaturated in short exposures and long exposures saturated Didymos to image the relatively faint ejecta and tail. All images were collected through filter F350LP (pivot wavelength 587 nm, bandwidth 149 nm)^30^.

The observations were planned to track at the Dimorphos ephemeris rate. The tracking nominally included corrections for parallax because of the orbit of the HST around Earth and was expected to keep Didymos inside the field of view with minimal drift in the field of view for all exposures. However, owing to an as-yet unexplained tracking problem, some orbits lost the target in various numbers of exposures, and some long exposures included a pointing drift of more than ten pixels. We limited our analysis to those exposures with fewer than seven pixels of drift and occasionally used long exposures with more drift when no good images were available for the particular orbits.

Images were calibrated by the HST standard calibration pipeline at the Space Telescope Science Institute^31^. We then removed the sky background measured from a square 100–400 pixels wide and 100–300 pixels from the top right corner, depending on the image size. This area is in general 20 arcseconds away from Didymos and shows no sign of any ejecta.

Aperture photometry was measured in all short, unsaturated exposures that have been corrected for charge transfer efficiency^31^ but not geometric distortion (.flc files, available from the HST data archive; see ‘Data availability’). The centroid was defined by a two-dimensional Gaussian fit with a 5 × 5 pixel box centred at the photocentre. The pixel area map was used to correct pixel area variations in the image^31^. The total counts were measured with circular apertures of a radius of 1–130 pixels (0.04–5.2 arcseconds). We converted the total counts to flux density and Vega magnitude based on the photometric calibration constants (PHOTFLAM = 5.3469 × 10^−20^ erg Å^−1^ cm^−2^ per electron, PHOTZPT = 26.78) provided in the image headers and the HST photometric calibration website. The total brightness of Didymos including the ejecta and the total brightness of ejecta are shown in Extended Data Fig. 4.

We used the images corrected for charge transfer efficiency and geometric distortion (.drc files) to study the morphology of the ejecta. To increase the signal-to-noise ratio of the faint ejecta features, we stacked all long exposures in each orbit because no change is visible in the ejecta morphology with each orbit. The centroid of long exposures that are saturated in the centre was determined by the cross-section of the diffraction spikes. Some long exposures with pointing-induced drift were included in the stack, but those with more than ten pixels of drift were discarded. The effects of this drift are accounted for as extra positional uncertainties to the measurements of features, which are mostly larger than the length of the drift. Cosmic rays and background stars were removed in the stacking process. Because different numbers of good long exposures were available for each orbit, the total exposure times varied from 25 to 50 s in most stacked long exposures and reached 155 s for the orbit 21 stack and 110 s for the orbit 23 stack.

Various image-enhancement techniques commonly used for studies of comets^32^ were used to assist the identification of ejecta features, including azimuthal median subtraction and division, azimuthal and radial reprojection, different brightness stretching and the use of various colour tables. All identified features were confirmed by several techniques.

The speeds of features as projected in the image plane were estimated by assuming that all features originated from the asteroid at the time of impact and moved directly away from the asteroid. The projected distance of a feature from the asteroid and the corresponding observation time yielded the projected speed of the feature. Note that the speeds estimated this way do not represent the true terminal speeds of the features after escaping the binary system for slow ejecta (< about 1 m s^−1^) or for features affected by solar radiation pressure. The trajectories of the features in those cases are notably affected by the gravity of Didymos (Extended Data Fig. 2) or solar radiation pressure.

### Ejecta cone opening angle and direction

We based our ejecta cone characteristics on the ejecta structures moving at more than 1 m s^−1^ in the images within *T* + 8.2 h (Fig. 2). These structures showed a linear motion moving away from the asteroid along the radial direction from the binary asteroid (Extended Data Table 2). Assuming that most of the ejecta dust is within a thin cone-shaped curtain, the two edges of the cone would appear as two bright rays along the radial direction from Dimorphos because of the optical depth effect when viewed from the side. Because the DART impact velocity is close to the sky plane (Extended Data Table 1), if we assume that the cone direction is close to the inverse of the DART impact velocity direction, the cone is close to being viewed from the side in the HST images and the opening angle spanned by the two edges of the cone (linear features l7 and l8) is close to its three-dimensional opening angle. This is confirmed by our derived cone geometry described in the following.

We measured the position angles of the two edges of the ejecta cone from both the original and the enhanced images (see ‘Observations and data reduction and processing’). The uncertainty range of the position angles is defined by the apparent width of the linear feature. Our measurement resulted in an ejecta cone centred within 5º of the incoming direction of DART with an opening angle of about 130º. Because of the fuzziness of the ejecta rays and their slight curvature, the uncertainty of the measured position angles could be as high as ±8º, resulting in an uncertainty of the opening angles up to ±12º. Taking the mean of these two edges and the maximum value of the uncertainty yields the ejecta cone axis at a position angle of 67° ± 8° under the assumption that the ejecta cone is axisymmetric about the cone axis.

To further constrain the ejecta cone geometry, we constructed a three-dimensional numerical cone model parameterized by the direction of the cone axis in right ascension (RA) and declination (dec), as well as an opening angle, to compare with the images. We first projected the six early post-impact images (Fig. 2) in an azimuthal–radial projection and, for each image, generated a histogram of pixels brighter than 18 mag arcsec^−2^ along the azimuthal direction. The azimuthal bins with the highest pixel counts (except for those of the tail and diffraction spikes) define the two cone edges with approximately a Gaussian distribution. The mean and the 1*σ* uncertainty of the position angles of the two cone edges are derived from the histograms. On average, the northern and southern cone edges are at position angles of 4º ± 8º and 131º ± 8º, respectively, consistent with the measurements described above. We then generated simulated images from the model ejecta cone and computed the corresponding histogram following the same approach for the actual images. This histogram was compared with the measured cone edge position angles to calculate a score, defined as

$$f\,=\mathop{\sum }\limits_{i=1}^{2}\mathop{\sum }\limits_{j=1}^{n}\frac{{s}_{j}}{{\sigma }_{i}\sqrt{2\pi }}\exp \left(-\frac{1}{2}\frac{{({x}_{j}-{\mu }_{i})}^{2}}{{{\sigma }_{i}}^{2}}\right)$$

where *σ*_*i*_ and *μ*_*i*_ are the standard deviation and mean of the northern or southern edges (*i* = 1, 2), respectively, *x*_*j*_ is the position angle of the histogram bin *j* for the simulated image and *s*_*j*_ is the pixel count in bin *j*. We searched the cone axis in the full range of RA and dec and the opening angle in 100º–160º for the highest score. Because the HST images alone could not determine whether the cone faced towards or away from Earth, this approach resulted in a pair of best-fit cone-axis solutions that were symmetric with respect to the image plane. We thus considered both as feasible cone-axis directions. The uncertainties of the solutions were estimated with 500 random samples of the measured cone edge position angles distributed in two Gaussians with the measured means and standard deviations. The best-fit cone-axis directions were (RA, dec) = (141º ± 8º, 25º ± 6º) and (120º ± 9º, 10º ± 7º), both with an opening angle of 125º ± 10º (1*σ* uncertainties). Both solutions are about 12º from the image plane, with the former pointing towards Earth and the latter pointing away.

### Dynamic model of the tail

The position angle of the tail and its uncertainty were determined by the radial directions from the asteroid that define the visible boundary of the tail at the furthest point along the tail in all (short and long exposures) stacked images that contain the tail. The dust dynamics model under the influence of solar radiation pressure follows a previous model^22^, in which the motion of dust is determined by *β*_srp_. *β*_srp_, which is defined as the ratio of the solar radiation pressure force to the solar gravitational force, depends on particle radius, *r*, and density, *ρ*, as

$${\beta }_{{\rm{srp}}}=K{Q}_{{\rm{pr}}}/\rho r$$

where *K* = 5.7 ×  10^−4^ kg m^−2^ is a constant, *Q*_pr_ is the radiation pressure coefficient averaged over the solar spectrum, which is usually assumed to be 1. We assumed a grain density of 3.5 × 10^3^ kg m^−3^ for the dust in the ejecta, following the density of ordinary chondrite meteorites^33^, considering that the Didymos–Dimorphos system shows an S-type spectrum that is associated with LL ordinary chondrite material^34^.

Pre-impact modelling suggested that the acceleration of solar radiation pressure always exceeds that of the gravitational acceleration of the Didymos system for ejecta particles smaller than 100 µm in size^20,35^. These small particles are pushed out of the binary system in less than 10 h. The gravity of Didymos is predominant within about 3 km for millimetre-sized particles and 10 km for centimetre-sized particles.

**Extended Data Fig. 7:**
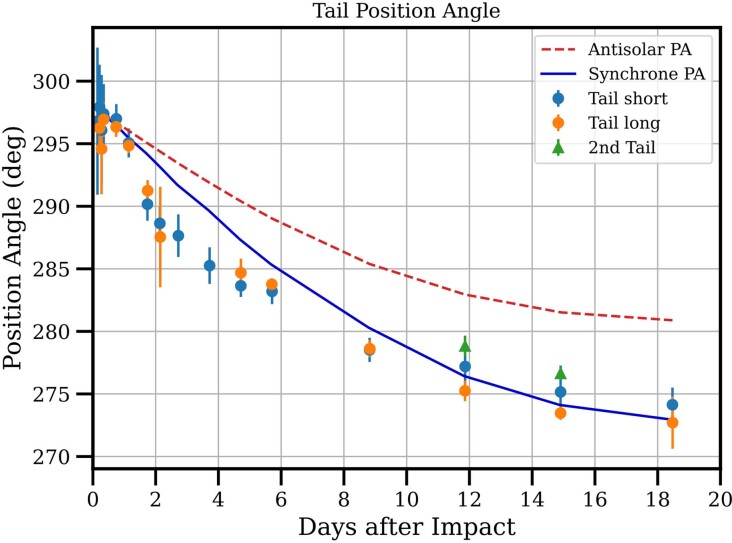
The position angles of the tail measured from HST images. The blue circles are measured from the stacked images of the short exposures, and the orange circles are measured from the stacked images from the long exposures. The green triangles are the position angles of the secondary tail. The red dashed line is the antisolar direction, and the blue solid line is the position angle of synchrones for dust emitted at the time of impact. The tail orientation measured from the short exposures could be affected by the secondary tail due to the low signal-to-noise compared to the long exposures.

The modelling of the orientation of the tail in the sky plane follows the synchrone–syndyne approach^36^, in which synchrones are the loci of dust particles ejected with zero initial velocity at the same time but with various *β*_srp_. The measured position angles of the tail of Dimorphos coincide to within 4º of the direction suggested by the synchrones associated with the time of impact in all images, suggesting that solar radiation pressure dominates the tail formation (Extended Data Fig. 7). The small discrepancy between *T* + 1 days and *T* + 5 days is probably because of the slight apparent curvature of the tail (Fig. 4e–h), which may be related to the non-zero mean initial velocity of dust particles with respect to the binary system, inherited from the orbital speed of Dimorphos.

The non-zero initial velocity of ejecta dust causes the tail to widen. The average initial velocity of the ejecta of Dimorphos, as projected in the image plane, has a northward component, which causes the tail to widen towards the north with respect to the loci of the hypothetical zero-velocity particle (synchrone). The relatively sharp southern edge and the more diffuse northern edge are consistent with the expectation from the ejecta mass–speed relationship^37^ because the number of dust particles decreases with increasing ejection speeds. The 1-arcsecond width of the tail is consistent with an initial velocity dispersion Δ*v* = 0.15 m s^−1^, comparable with the orbital speed of Dimorphos, suggesting that the tail is primarily composed of the slowest ejecta.

The inverse proportionality of *β*_srp_ with particle size means that small particles experience stronger solar radiation pressure and are pushed away from the asteroid faster after ejection than large particles. Because the duration of our HST observations is much shorter than the orbital period of Didymos around the Sun (2.1 years), the motion of particles along the tail relative to the asteroid under solar radiation pressure can be approximated by a constant acceleration motion. As the length of the tail grows, particles of various sizes spread out along the tail, with the smallest particles remaining near the far end of the tail from the asteroid and the larger particles dominating the end near the asteroid. Assuming a power-law differential particle-size distribution with an exponent of *α* for the tail, we derived that the brightness of the tail is expected to have a power-law relationship with the distance to the asteroid with an exponent *b* = −4 − *α*.

We extracted the brightness profiles of the tail from stacked long exposures from *T* + 5 h until the last stack at *T* + 18.5 days (Extended Data Fig. 3). The exponent *α* of the differential particle-size distribution was derived from the linear part of the tail-brightness profiles (in log–log space) in various images, corresponding to a range of *β*_srp_ from 0.2 × 10^−4^ to 8 × 10^−4^, to be between −2.2 and −3.1, with an average of −2.7 and a standard deviation of 0.2. The range of *β*_srp_ indicates that particle sizes range from 1 µm to a few millimetres. In images after about *T* + 6 days, the tail brightness displays two regions with different power-law slopes. The inner region appears to be influenced by the particles in the curved ejecta streams that started to overlap with the tail. The outer region has best-fit slopes close to −2.7 as in the early images, whereas the slope of the inner region ranges from −3.6 to −3.9. The range of *β*_srp_ for the inner region is 7 × 10^−4^ to 1 × 10^−5^, corresponding to millimetre- to centimetre-sized particles. The lack of small particles in the curved streams is expected because particles with a size of 100 µm or smaller should have been removed a few hours after the impact. The apparent increasing steepness of the particle-size distribution in this size range also seems to indicate that the bulk of ejecta particles have a size cut-off of a few centimetres. If the particle-size distribution of the tail represents that of all ejecta, then a power-law index of −2.7 means that the total ejecta mass is dominated by the largest particles.

The above treatment assumes that the albedo is independent of particle size, which needs to be examined. On the basis of laboratory measurements of the phase function of micromtre-sized aerosols^38^ and millimetre-sized particles^39^, along with supporting models of scattering efficiency^40^, the albedo of micrometre-sized particles is about 70% that of millimetre-size-grains at the phase angle of our early observations (54º). This brightness ratio is reversed at the phase angle corresponding to the final images (74º), in which micrometre-sized particles become about 16% brighter. Our calculation indicates that the small difference between the albedos of micrometre- and millimetre-sized particles changes the best-fit power-law index of the particle-size distribution by less than 2%. Our assumption of the same albedo for micrometre-to centimetre-sized particles holds.

### Secondary tail

The small decrease in the overall fading rate of the total brightness of the Didymos–Dimorphos system between about *T* + 5 days and *T* + 7 days indicates an increase in the total scattering cross-section in the ejecta within 10 km of the system (Extended Data Fig. 4), partly compensating for the ejecta moving out of the photometric aperture. It is unlikely to be caused by a change in albedo for the ejecta particles. Injection of new dust particles into the ejecta was considered.

This scenario and its timing are also supported by the synchrone model (Extended Data Fig. 6), in which the projected direction of the secondary tail is consistent with the synchrones associated with about *T* + 5.0 days to *T* + 7.1 days. The narrow width of the secondary tail similar to that of the original tail suggests a low initial velocity of about 0.15 m s^−1^ for the dust particles. Although the Didymos binary environment could complicate the dust motion and cause deviation from the zero initial velocity assumption of the idealized synchrone model, the observed low initial velocity of the dust in the secondary tail implies limited effects.

The possible mechanisms of the secondary dust emission could include the re-impact of ejecta blocks onto Dimorphos or Didymos^35^ or large ejecta blocks disintegrating into small pieces because of spin-up or mutual collisions (S.L.I. et al., manuscript in preparation). Mass shedding from the surface of Dimorphos because of rotation is not likely given its slow rotation if its spin is tidally locked. However, mass movement and shedding from Didymos could potentially be triggered by ejecta re-impact because its fast rotation causes a net outward acceleration at its equator, although no clear indication of this has been confirmed yet^3^. Once the dust is lifted from the surface of Dimorphos or Didymos using these mechanisms, solar radiation pressure will quickly sweep the dust into the antisunward direction, forming a secondary tail.

Other mechanisms, such as the dynamic interaction between the slow ejecta dust and the binary system^41^, gravitational scattering for the ejecta dust when they are turned back by solar radiation pressure and pass the binary system, or photon-charged dust particles under the influence of interplanetary magnetic field^42^ could also result in the unusual tail morphology that leads to the appearance of a secondary tail. Our dynamics simulations suggested that a secondary dust emission is not necessary to form a secondary tail that has a morphology consistent with the one observed. However, these scenarios may not be accompanied by the increase in ejecta dust as suggested by the fading lightcurve of the Didymos system.

## Online content

Any methods, additional references, Nature Portfolio reporting summaries, source data, extended data, supplementary information, acknowledgements, peer review information; details of author contributions and competing interests; and statements of data and code availability are available at 10.1038/s41586-023-05811-4.

### Supplementary information

Supplementary Video 1 Animation of the HST image sequence of evolution of ejecta from Dimorphos. In all frames, north is up and east to the left—that is, the same orientation as all of the figures in the Article. All frames are displayed with the same logarithmic brightness scale.

## Figures and Tables

**Extended Data Table 1:**
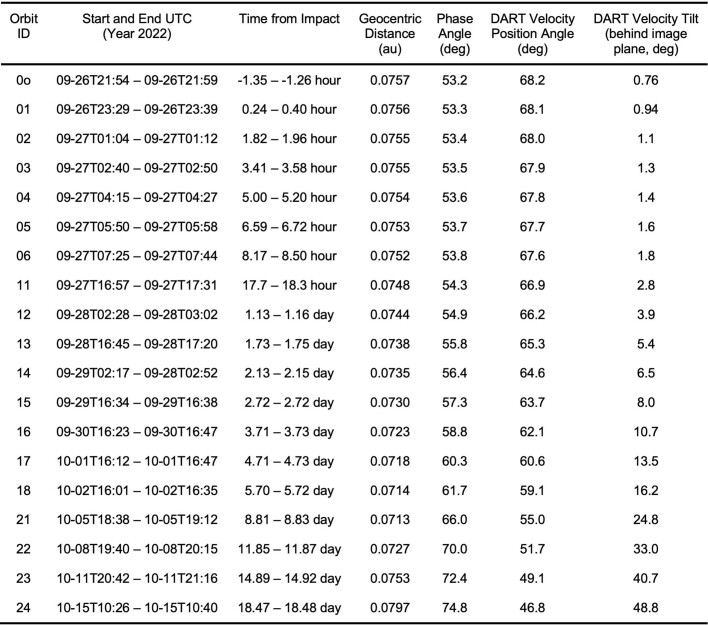
HST observations of DART impact

This table lists the times and observing geometries for each of the 19 orbits of HST observations. The parameters listed in this table only refer to usable images in each orbit. Some images were lost due to tracking problems.

**Extended Data Table 2:**
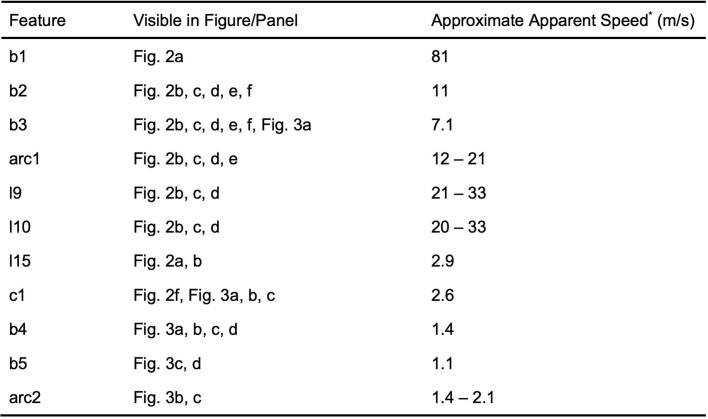
Selected features and their approximate plane of sky speed

The speeds reported here are averaged over all measurements in multiple images for every feature. The scatters in measured speeds are typically < 5% for each feature. The measurement is based on the approximate distance of the feature to the asteroid and the corresponding mid-observation times.

## Data Availability

All raw HST data associated with this Article are archived and are publicly available at the Mikulski Archive for Space Telescopes (https://mast.stsci.edu/search/ui/#/hst/results?proposal_id=16674) hosted by the Space Telescope Science Institute. The stacked long exposures in Figs. 2–4 are available from a website hosted at JHU/APL (https://lib.jhuapl.edu/papers/ejecta-from-the-dart-produced-active-asteroid-dimo). Other related data are available from the corresponding author upon request.
